# Atlantic multi-decadal oscillation covaries with Agulhas leakage

**DOI:** 10.1038/ncomms10082

**Published:** 2015-12-10

**Authors:** Arne Biastoch, Jonathan V. Durgadoo, Adele K. Morrison, Erik van Sebille, Wilbert Weijer, Stephen M. Griffies

**Affiliations:** 1 GEOMAR Helmholtz Centre for Ocean Research Kiel, Düsternbrooker Weg 20, 24105 Kiel, Germany; 2 Atmospheric and Oceanic Sciences Program, Princeton University, 300 Forrestal Road, Princeton, New Jersey 08544, USA; 3 Climate Change Research Centre & ARC Centre of Excellence for Climate System Science, University of New South Wales, Sydney, New South Wales 2010, Australia; 4 Grantham Institute & Department of Physics, Imperial College London, London SW7 2AZ, UK; 5 Los Alamos National Laboratory, Los Alamos, New Mexico 87545, USA; 6 NOAA Geophysical Fluid Dynamics Lab, 201 Forrestal Road, Princeton, New Jersey 08542, USA

## Abstract

The interoceanic transfer of seawater between the Indian Ocean and the Atlantic, ‘Agulhas leakage', forms a choke point for the overturning circulation in the global ocean. Here, by combining output from a series of high-resolution ocean and climate models with *in situ* and satellite observations, we construct a time series of Agulhas leakage for the period 1870–2014. The time series demonstrates the impact of Southern Hemisphere westerlies on decadal timescales. Agulhas leakage shows a correlation with the Atlantic Multi-decadal Oscillation on multi-decadal timescales; the former leading by 15 years. This is relevant for climate in the North Atlantic.

## Abstract (web summary)

The long-term quantification of the Angulas Leakage is difficult due to its highly variable spatio-temporal nature and sparse observations. Here, the authors combine sea surface temperature with a series of ocean and climate model simulations to construct a 145-year long time series of Agulhas leakage.

## Graphical abstract

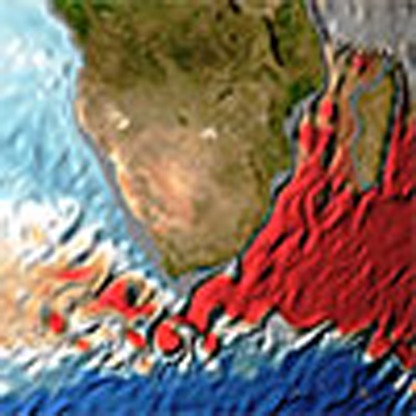

The global overturning circulation (GOC) is key for the oceanic redistribution of heat and carbon, and influences global climate^1^. Along its downwelling limbs, anthropogenic trace gases are removed from the atmosphere and stored in the deep ocean on timescales of centuries to millennia. In-depth understanding of the driving mechanisms of the GOC and its temporal evolution are thus fundamental goals of climate science research. Since the original ‘Conveyor Belt' concept^2^, the scheme of the GOC has been redrawn and refined, now revealing less coherent pathways for both upper and deeper limbs^3^,^4^. Despite a more complex picture, key regions still exist where the GOC converges, and where regionally constrained and small-scale processes can have a large-scale impact. Such choke points include passages through the Indonesian Throughflow and the Agulhas region for the upper branch and gaps in the Greenland–Scotland Ridge for the deeper branch.

In the Agulhas region around South Africa, part of the return flow of surface and intermediate waters from the Indian Ocean leaks into the Atlantic, providing heat and salt that is advected towards the deep-water formation regions of the subpolar and subarctic North Atlantic^5^. The Agulhas Current^6^ flows poleward as a western boundary current and abruptly turns eastward into the Agulhas Return Current. In so-doing, Agulhas rings are shed; a process that is modulated by upstream mesoscale features, resulting in an episodic interoceanic transport^7^. Models suggest that the amount of Agulhas Current waters ending up in the Atlantic Ocean, ‘Agulhas leakage', strongly varies on inter-annual to decadal timescales^8^.

Despite the regional convergence of the GOC south of Africa, Agulhas leakage is not easily quantifiable with ocean observations due to its highly variable spatio-temporal character^7^. Ocean observations of this inter-ocean flow with surface drifters and subsurface floats only provide a rough time-mean estimate of 15 Sv (1 Sv=10^6^ m^3^ s^−1^)^9^. Agulhas leakage has additionally been estimated using surface circulation from satellite altimetry for the past two decades^10^,^11^, and using a decade of hydrography collected by autonomous floats^12^. An upward trend in eddy kinetic energy based on sea surface height (SSH) in the Cape Basin has been used to argue for an increase in Agulhas leakage^13^. However, it has also been pointed out that the evolution of the non-eddying component is an important contribution to Agulhas leakage; its trend is much more unclear^14^. Because of its climatic relevance, a long-term time series is desirable, but cannot be obtained from the sparsely observed three-dimensional (3D) description of velocity and hydrography around southern Africa. However, while the full-depth structure of temperature is not available, the surface component is. Due to its easy access, from simple bucket to modern satellite measurements, sea surface temperature (SST) compilations provide one of the longest observational records available for the global ocean.

Here we combine SST with a series of ocean and climate model simulations to construct a 145-year-long time series of Agulhas leakage. We show that Agulhas leakage is decadally modulated by wind-driven dynamics in the Southern Hemisphere and that the leakage covaries with a key mode of climate variability in the North Atlantic.

## Results

### Agulhas leakage imprint on SST

**Figure 1:**
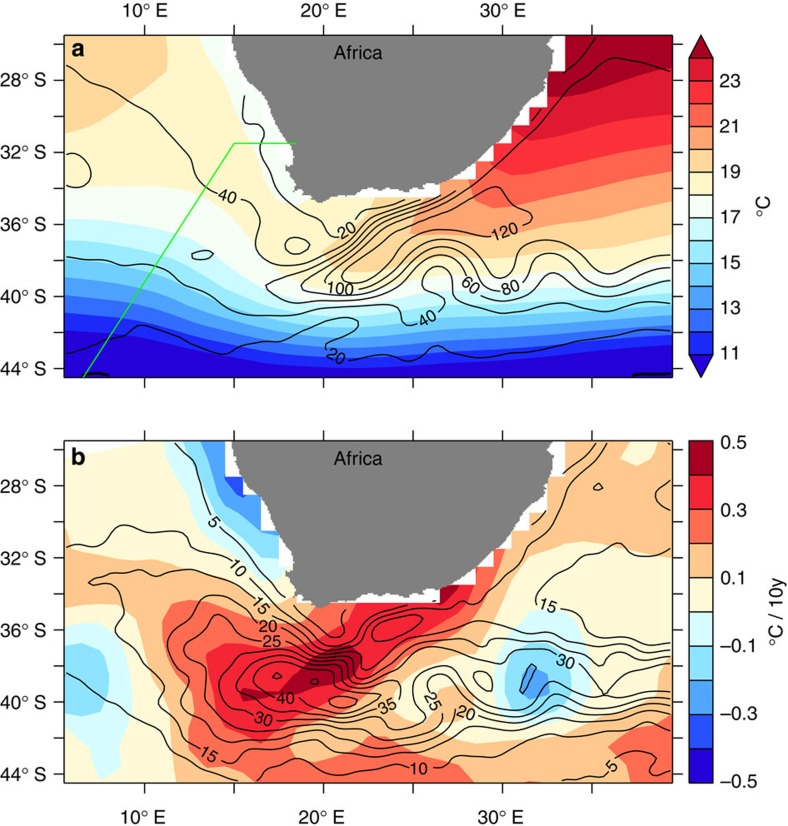
SST shows decreasing contrast between the Indian Ocean and Atlantic. (**a**) Time-mean observed SST (1965–2000; colour) and SSH (1993–2013; contoured, in cm). (**b**) Linear SST trend (1965–2000; colour) and SSH s.d. (contoured, in cm). The green line in **a** shows the section used for the Lagrangian Agulhas leakage calculation.

We use the Hadley Centre SST data set (HadISST)^15^, available for the period 1870–2014. Maps from this data set illustrate well the clear temperature contrast between the two adjacent oceans, with warmer Indian Ocean water leaking into the relatively colder South Atlantic (Fig. 1a). Pairing this information with SSH from satellite altimetry demonstrates a close relation between SST and circulation, in particular in delineating the Agulhas Current and Agulhas leakage. The temporal evolution of SST provides insight into the long-term changes in the region. Consistent with other observational studies^16^, the Agulhas Current and Agulhas ring corridor exhibit a strong warming signal of up to 0.4 °C per decade, whereby the southwest Indian Ocean shows a cooling pattern (despite global warming) in the recent decades (Fig. 1b). We investigate whether this reduction of the temperature gradient between the two oceans reflects a redistribution of heat, caused by additional inflow of Agulhas waters into the Atlantic.

To test this hypothesis, we use output from a series of model simulations. Since the accurate representation of the mesoscale flow and the quantification of Agulhas leakage require sufficient resolution^17^, we concentrate on the 1/10° ocean models INALT01 (ref. 17) and OFES^18^. Both have been run in hindcast mode, in which the atmospheric forcing consists of observationally derived data from the past 50–60 years. The modelled SST and SSH mean distributions, temporal variability, and trends over the past decades resemble the observations (Supplementary Fig. 1). It is important to note that the upward trend from the mid-1960s to the 2000s in HadISST is well matched by INALT01 and OFES (Supplementary Fig. 2). Since SST in ocean-only models follows the prescribed air temperature to a certain degree, we use the near-surface temperature (NST) at ∼15 m depth. To test potential limitations in this approach, we include SST simulated by a modern climate model at similar ocean resolution. Geophysical Fluid Dynamics Laboratory's (GFDL) Climate Model version 2.6 (CM2.6)^19^ is coupled to an active atmosphere and simulates its own variability under prescribed preindustrial CO_2_ concentration. Within the time-varying flow, we quantify Agulhas leakage at annual resolution using a Lagrangian approach^8^,^20^ by following water parcels from the Agulhas Current into the South Atlantic (green line in Fig. 1a) using the Connectivity Modeling System (CMS)^21^.

**Figure 2:**
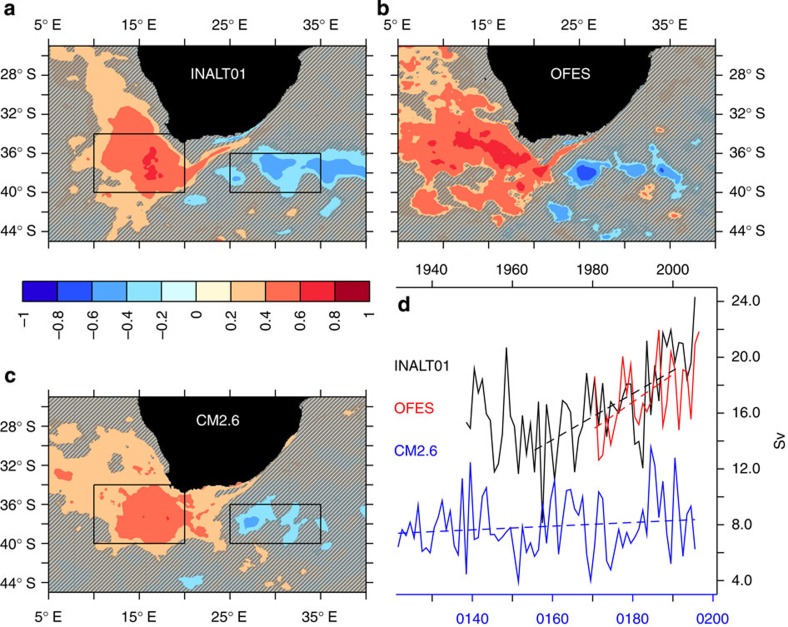
Agulhas leakage and SST are highly linked around South Africa. Correlation between annual Agulhas leakage and NST for the forced-ocean models (**a**) INALT01, (**b**) OFES and (**c**) SST for the coupled climate model CM2.6 (regions hashed in grey denote significance below the 99% level). (**d**) Annual Agulhas leakage time series (dashed lines show linear trends, with INALT01 (OFES) significant at the 99% (95%) level and CM2.6 not being significant). Rectangles in **a**–**c** show regions where NST and SST are averaged.

All models show significant correlation between Agulhas leakage and SST/NST, with years of high leakage corresponding to higher temperature in the Cape Basin and along the path of Agulhas rings, and colder temperature in the southwest Indian Ocean and along the Agulhas Return Current (Fig. 2a–c). All models employed here resolve the nonlinear flows associated with Agulhas leakage. This is evident in the strong year-to-year variability of leakage and its spread into the Atlantic (Fig. 2d). Although individual years do not match because of the internal variability and different forcing products, both INALT01 and OFES simulate a similar upward trend in the last decades, in contrast to no discernable trend due to the stable radiative forcing (by construction to a fixed CO_2_ level) within CM2.6 (Fig. 2d).

To underline the importance of ocean dynamics on the correlation, Supplementary Fig. 3a,b presents the correlation between Agulhas leakage and temperature at 100 m depth. Away from the direct atmospheric influence (both in the forced-ocean model as well as in the atmospherically coupled model), the pattern of positive correlation in the Cape Basin and negative correlation along the Agulhas Return Current in the Indian Ocean still holds. The importance of ocean dynamics is also given by the fact that no significant correlation exists between the air temperatures used to force INALT01 and Agulhas leakage (Supplementary Fig. 3c).

### A 145-year-long time series of Agulhas leakage

**Figure 3:**
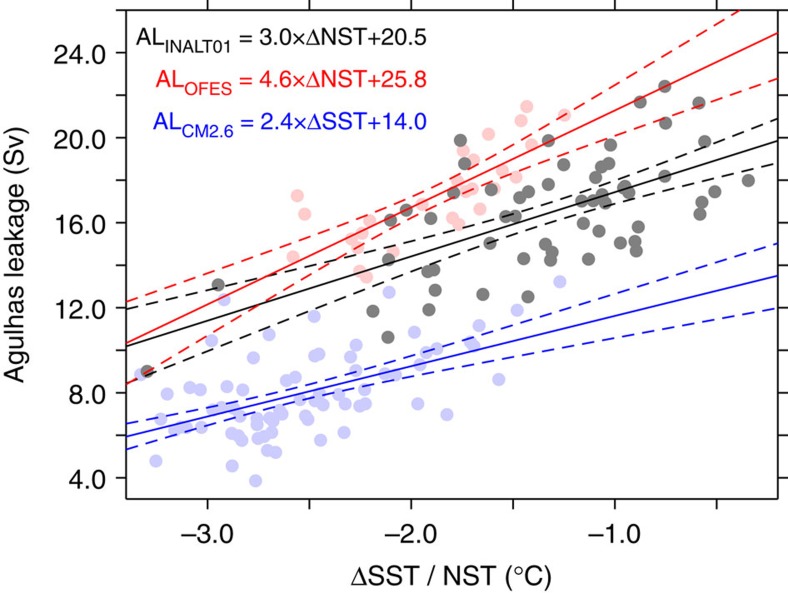
Regressions. Annual Agulhas leakage versus SST/NST (Atlantic Ocean minus Indian Ocean, for averaging boxes see Fig. 2) for INALT01 (black), OFES (red) and CM2.6 (blue) with linear fits (thick lines and formula) and 95% confidence intervals (dashed lines). (Note that the time series were detrended before the regression and that the regression holds for the range of temperature difference).

The high correlation within each of the individual models allows us to regress Agulhas leakage from HadISST. The linear relationships between Agulhas leakage and the gradient of SST/NST for the three models are shown in Fig. 3.

**Figure 4:**
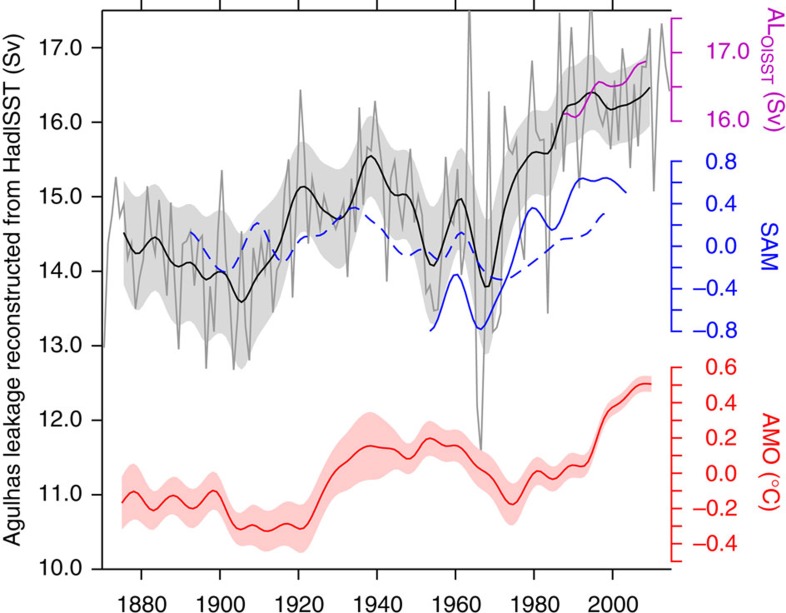
A 145-year-long historic time series of Agulhas leakage. Annual (grey) and decadally filtered (black) Agulhas leakage (AL) regressed from HadISST and from OISST (purple) using the INALT01 relation (shading shows the 95% confidence interval from the regression, Fig. 3); decadally filtered SAM indices from reanalysis^22^ (blue solid, correlation with AL (both detrended), *r*=0.89*) and historic analysis^23^ (blue dashed, *r*=0.59**). Agulhas leakage and AMO index (red, HadISST anomaly, averaged between 0°–60° N and 75°–7.5° W) are lag-correlated (*r*=0.74**) by 15 years (significant at *99%, **95% confidence interval, data detrended. Note that the correlation also holds if the global mean temperature is removed instead of detrending the AMO time series). The AMO uncertainty was estimated using the square mean difference between HadISST and an independent data set (ERSSTv3b (ref. 45)).

The reconstructed 145-year-long time series of Agulhas leakage is based on the regression from INALT01. It shows a strong year-to-year variability, with prominent decadal variations, and a trend of 0.16 Sv per decade over the entire period (Fig. 4). The salient upward swing of 0.84 Sv per decade from the mid-1960s is consistent, but smaller than the one simulated by INALT01 (1.7 Sv per decade, Fig. 2d, all trends significant at the 99% level). While the trends in temperature gradients are roughly comparable (Supplementary Fig. 2), the regressions from the individual models differ (Fig. 3), explaining differences of up to 50% in the trends for the period 1965–2000 (Supplementary Fig. 4b). Using the regression from OFES and CM2.6 alters the absolute numbers, but not the temporal evolution of the regressed Agulhas leakage (Supplementary Fig. 4b), as this is determined by the temperature gradient (Supplementary Fig. 2). The determination of the regressed Agulhas leakage from HadISST depends on the gradient between the Cape Basin and the Indian Ocean (boxes in Fig. 2a–c), not on the individual regions itself (Supplementary Fig. 4a).

### Control and impact of Agulhas leakage

The increasing trend of Agulhas leakage since 1965 in INALT01 was attributed to intensifying Southern Hemisphere westerlies^17^. Since westerlies essentially resemble the atmospheric pressure difference between the subtropics and Antarctica, the Southern Annual Mode (SAM) is decadally correlated with Agulhas leakage. This relationship, which has so far only been indicated by ocean models^14^, is now independently confirmed by observations. The regressed time series of Agulhas leakage based on HadISST shows a strong correlation with SAM in the well-observed recent decades, for example, from reanalysis data^22^; a correlation is also evident in a historic compilation^23^. From the evolution of the regressed Agulhas leakage post-2000s, it seems that Agulhas leakage stabilizes, consistent with an independent analysis using satellite altimetry^11^ (Supplementary Fig. 5). The alternative OISST data set^24^, however, predicts a continuation of the increase (Fig. 4), similar to model experiments with strengthening westerlies as a result of global warming^25^.

One important relevance of Agulhas leakage is its potential impact on the GOC^5^. North Atlantic SST, represented by the Atlantic Multi-decadal Oscillation (AMO), serves as a proxy for internal variability of the GOC^26^, with widespread implications for Northern Hemisphere climate^27^, including regional phenomena such as hurricanes and droughts. It is interesting to note that the reconstructed Agulhas leakage and AMO are correlated on multi-decadal timescales (Fig. 4), with Agulhas leakage leading by 15 years. This timescale is consistent with transit time analyses of Agulhas leakage into the North Atlantic^28^. It is to note that the relation between Agulhas leakage and AMO is restricted to the Atlantic Ocean; it also holds if the global mean temperature is removed from the SSTs before the calculation of the correlation.

**Figure 5:**
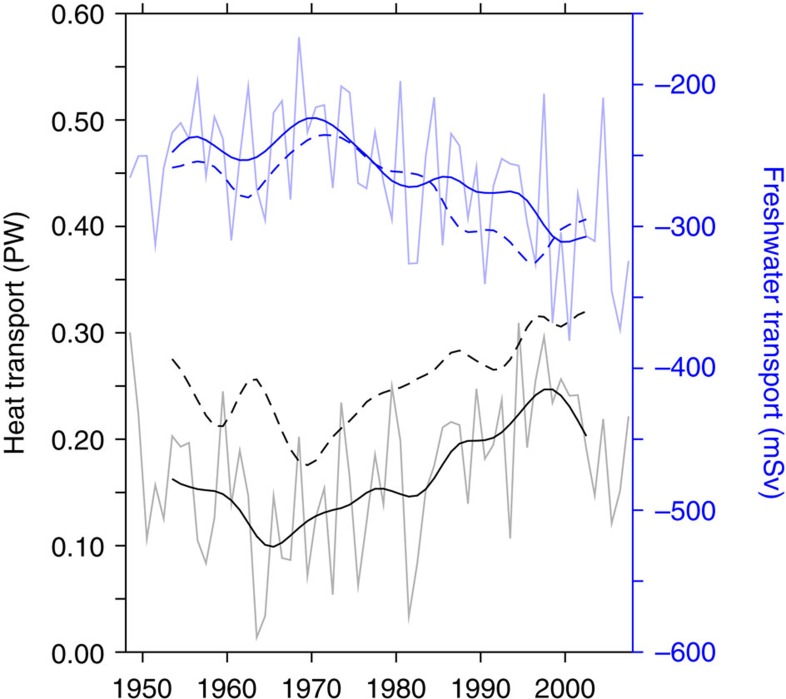
Heat and salt transport of Agulhas leakage. Heat (black) and freshwater transports (blue, referenced to *S*=35, negative values indicating salt transport) in INALT01 through Agulhas leakage (solid lines) and as meridional transports in the South Atlantic (10°–5° S average, dashed lines).

**Figure 6:**
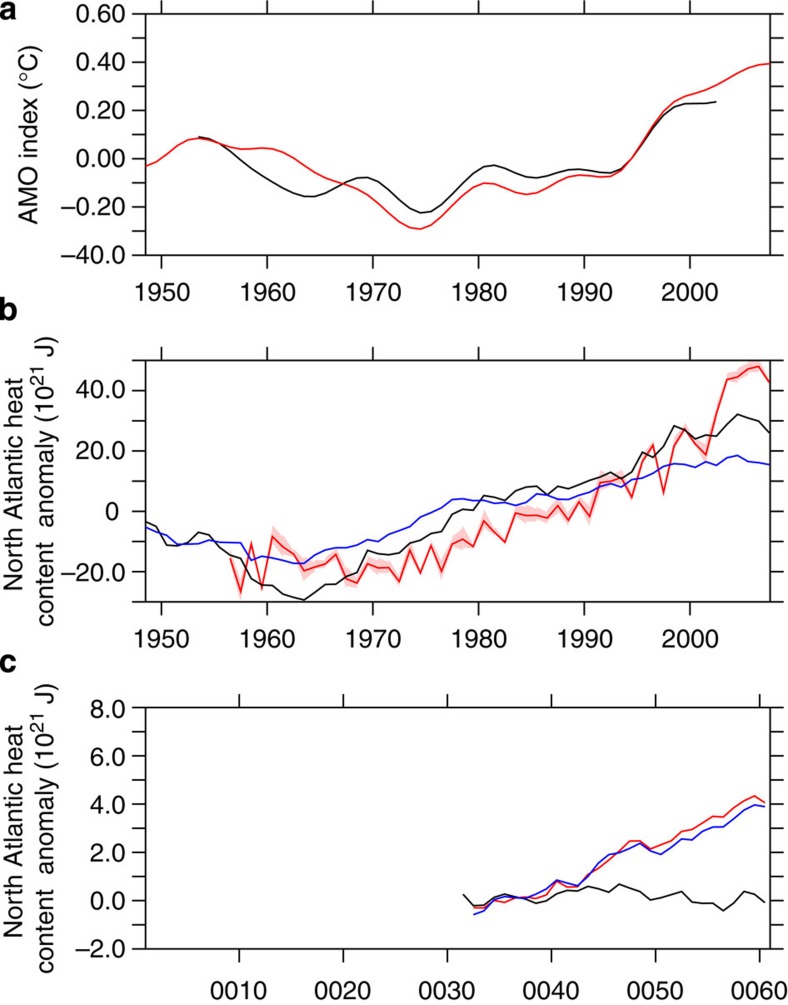
Sensitivity experiment. (**a**) AMO index in the INALT01 hindcast (black) and HadISST (red, as in Fig. 4). (**b**) North Atlantic (including Arctic Ocean, as in ref. 30) heat content anomaly in the INALT01 hindcast, integrated between 0–700 m (black) and 200–700 m (blue) and from observations (0–700 m)^30^ (red shading denotes the observational error). (**c**) North Atlantic heat content in INALT01 repeated-year forcing experiments: black reference, red (0–700 m) and blue (200–700 m) the sensitivity experiment with artificially isolated increase in Agulhas leakage. All experiments were linearly detrended using the repeated-year forcing experiment.

Despite the limitations of INALT01 and OFES as forced-ocean models, they provide the potential of hindcast simulations. The modelled heat and freshwater transports by Agulhas leakage are clearly linked to heat and salt exports across the Atlantic (Fig. 5). Consistent with earlier studies with coarse-resolution models^29^, the anomalous meridional heat transport can explain the increase in North Atlantic heat content. AMO and heat content in INALT01 compare well to the observations^30^ (Fig. 6a,b).

In addition to Agulhas leakage, other processes such as local atmospheric forcing or subpolar North Atlantic dynamics also impact North Atlantic heat content. The potential impact of Agulhas leakage is isolated using a sensitivity experiment in which Agulhas leakage was artificially enhanced by 2.5 Sv (ref. 17). Both the control and perturbation runs were forced by repeated-year forcing. The heat transport of Agulhas leakage in the perturbed run is 0.28±0.03 PW, significantly higher (based on a Welch's *t*-test) than the 0.20±0.03 PW of heat transport in the reference experiment. 10–15 years after the increase in heat transport, the North Atlantic heat content starts to increase (Fig. 6c). In particular, the heat content between 200 and 700 m depth is most affected by Agulhas leakage. This is agreement with observed temperature changes in the upper tropical Atlantic Ocean which can be traced back to Agulhas leakage^31^. After 30 years, North Atlantic 200–700 m heat content has increased by 4 × 10^21^ J compared with the control. A simple scaling argument would suggest that the 6 Sv change in the hindcast experiment between the 1970s and 2000s (Fig. 2d) would correspond to a 9.6 × 10^21^ J increase in the North Atlantic 200–700 m heat content, if applied instantaneously. This number would in turn correspond to 40% of the simulated changes over the same depth range in the hindcast experiment between the 1970s and 2000s (Fig. 6b), and 20–25% of the changes if we consider the entire 0–700-m-depth range in both hindcast and observations^30^.

The sensitivity experiment suggests that Agulhas leakage can explain up to 20–40% of the observed^30^ North Atlantic heat content increase. We note that the sensitivity experiment is very idealized, with artificial changes in the volumetric Agulhas leakage only and no corresponding changes arising from the thermohaline atmospheric forcing or changing water masses. It would be natural to further isolate the mechanisms in the coupled model CM2.6. However, we speculate that the low transport of Agulhas leakage in CM2.6, paired with the weak simulated AMO (refs 19, 32), disrupts the relative importance between northern and southern influences on the GOC. This emphasizes the need to further improve high-resolution coupled climate models^19^,^33^.

## Discussion

In this study, we link Agulhas leakage to the SST contrast between the Indian Ocean and the South Atlantic. Motivated by correlations from a suite of ocean-only and coupled climate models we construct a 145-year-long time series based on observations. The regressed Agulhas leakage time series shows a multi-decadal variability and a concomitant long-term trend due to global warming. The decadal variability, which seems to have reached a plateau post 2000 is linked to wind-driven changes through Southern Hemisphere westerlies. On the multi-decadal timescale Agulhas leakage is linked with changes in heat and salt transports towards the North Atlantic, and in consequence, SST and heat content changes therein.

Although the sensitivity experiment with artificially increased Agulhas leakage can only explain part of the observed heat content increase in the North Atlantic, it provides a useful insight on the depth structure of Agulhas leakage and the expected signal in observations. The AMO is seen as an indicator of GOC changes, rather than a simple advection of SST from the Agulhas region towards the North Atlantic. The northward spreading Agulhas leakage occurs subsurface^31^, whereby the surface temperatures are in close contact with the local atmosphere. Agulhas leakage is certainly an important player for GOC changes in the North Atlantic, but it is certainly not the sole actor and should be also seen in concert with other effects from changing conditions in the subarctic and subpolar North Atlantic.

The specialized reader may have noticed that we did not discriminate the impact of Agulhas leakage on the GOC into thermohaline and wind-driven processes. Although it seems plausible that the long-term link that the Agulhas region provides is part of the global thermohaline circulation, for example, receives water masses from the Indonesian Throughflow^34^, the amount and the temporal variability of Agulhas leakage largely depends on the Southern Hemisphere westerlies^17^. Our study indicates that Agulhas leakage monitors a long-term increase of the GOC. On decadal (probably also multi-decadal) timescales, through respective wind changes in the Southern Hemisphere, it dynamically modulates the Atlantic imprint of the GOC. Dedicated experiments, in particular with high-resolution coupled climate models, that will become routinely feasible only in the coming years are required to provide deeper mechanistic insight, in particular to elucidate whether Agulhas leakage plays an active role in modulating northern hemisphere climate.

## Methods

### Ocean and climate model data

We used 3D, time-varying output from two ocean models (INALT01, OFES) and one coupled climate model (CM2.6), all at similar resolution of 9–11 km within the Agulhas region.

INALT01 (ref. 17) consists of a 1/10° nest covering the South Atlantic and western Indian Ocean (50° S–8° N, 70° W–70° E) and a global ocean/sea–ice model at ½° resolution^35^ based on the Nucleus for European Modelling of the Ocean (NEMO v3.1.1)^36^. Nest and base are linked through the Adaptive Grid Refinement in Fortran (AGRIF)^37^, providing boundary conditions to the nest and updating the base model in the nested region at every time step. The atmospheric forcing is provided by interannually varying data (1948–2009) from the Common Ocean–Ice Reference Experiments (CORE v2b)^22^ and applied through Bulk formulae at 6-h (momentum, heat and freshwater), daily (radiation) and monthly (rain and snow) resolution. INALT01 has been verified and used in a range of applications in the Agulhas region^17^,^28^,^38^,^39^. To remove a small model trend in Fig. 5, an experiment under repeated-year forcing was used. The impact of Agulhas leakage on the North Atlantic (Fig. 6) was isolated using experiment SHW+40% LOCAL from ref. 17.

Ocean General Circulation Model For the Earth Simulator (OFES)^18^ is a quasi-global (75°S–75°N) ocean model at 1/10° resolution, run at Japan Agency for Marine-Earth Science and Technology (JAMSTEC) and based on the Modular Ocean Model (MOM3) code. It is forced with a combination of satellite winds^40^ and NCEP/NCAR reanalysis data^41^, applied through Bulk formulae. Here, snapshots of 3D velocity every 3 days from the last 31 years of the run are used (from January 1980 to December 2010). OFES has been analysed in Agulhas-related dynamics^39^,^42^,^43^.

CM2.6 is the high-resolution version^19^ of the GFDL CM2-O coupled model suite. It combines a global 1/10° ocean/sea–ice model with 50 km versions for the atmosphere and land components. The ocean component is based on MOM5 code, and employs no eddy parameterizations for either eddy-induced tracer advection or neutral diffusion. A preindustrial control simulation is used with atmospheric CO_2_ concentration fixed at 286 p.p.m. CM2.6 is spun up from climatological tracer fields for 120 years preceding the period used for analysis here.

### Lagrangian quantification of Agulhas leakage

The quantification of Agulhas leakage follows a Lagrangian approach using the CMS (ref. 21). We employ an established strategy^8^,^11^,^17^,^28^ and continuously seed particles within a 300-km section of the Agulhas Current at 32° S. Each particle is allocated a partial transport, such that cumulatively they represent the initial poleward volume transport of the current. The particles are advected using the model's 3D, time-varying (3-daily mean for OFES, 5-daily mean for INALT01, monthly mean for CM2.6; note that the averaging period is of minor importance, Supplementary Fig. 6) velocity fields. The sum of the particles reaching a control section west of the retroflection (green line in Fig. 1a, close to the Goodhope section^44^) gives Agulhas leakage, the portion of Agulhas Current entering the South Atlantic, at annual resolution. Since the advection within the Agulhas Current towards the southern tip of Africa takes place within the first year, particles are considered as Agulhas leakage (and attributed to the year of release) when reaching the Goodhope section within the following 4 years (accounting for the nonlinear flow in the Cape Basin).

### Interoceanic heat and freshwater transports

South of Africa, the zero line of the barotropic streamfunction separates the Agulhas regime from the Antarctic Circumpolar Current. The full-depth integration, from the continent to the zero line of the streamfunction 
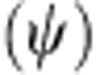
 along the Goodhope section, volumetrically closes the transport and allows us to integrate the product of velocity and temperature or salinity to infer heat and freshwater transports. Since the streamfunction varies over time, the integration (here shown for heat transport) is performed at monthly resolution:


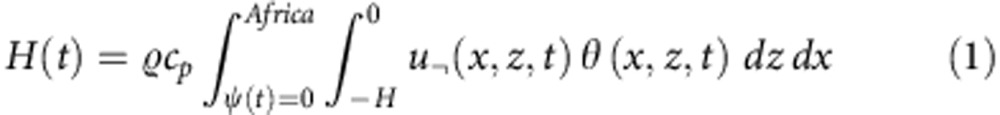


using the cross-component of velocity at Goodhope 
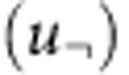
, potential temperature (*θ*) and density 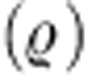 and specific heat capacity of seawater (*c*_*p*_).

### Statistics

All correlations and regressions were performed after removing linear trends. Statistical significances were calculated based on a Student's *t*-test considering effective degrees of freedom using the autocorrelations.

### Observational data

For the construction of the time series of Agulhas leakage we use SSTs from the HadISST historic compilation^15^, at 1° spatial and monthly temporal resolutions available for the period 1870–2014 at http://www.metoffice.gov.uk/hadobs/hadisst, and an independent combined satellite – *in situ* product OISST (ref. 24), available for the period 1982–2014 from http://www.esrl.noaa.gov/psd/data/gridded/data.noaa.oisst.v2.html. As an independent estimate for the AMO we used the ERSSTv3b (ref. 45) data set (http://www.esrl.noaa.gov/psd/data/gridded/data.noaa.ersst.html). Absolute SSH was obtained from satellite altimetry, available at ¼° spatial and daily resolution since 1993 from http://www.aviso.altimetry.fr.

SAM indices were calculated from the CORE data set^22^ as the difference of the normalized zonal mean sea-level pressure anomalies between 40° S and 65° S. For the long-term evolution, we used a historic compilation^23^, downloaded from http://www.geomar.de/~SAM, which was considered as reliable especially in the pre-satellite era^46^.

### Code availability

Code to run the CM2.6 experiment is available from http://www.gfdl.noaa.gov/cm2-5-and-flor. The website of the OFES project is http://www.jamstec.go.jp/esc/research/AtmOcn/product/ofes.html. INALT01 code and data are provided upon request. The CMS code is available from https://github.com/beatrixparis/connectivity-modeling-system. The 145-long reconstructed time series of Agulhas leakage is available at http://data.geomar.de/.

## Authors contributions

A.B. and J.V.D. conceived the analysis. E.v.S., J.V.D. and A.K.M. performed the CMS integrations for OFES, INALT01 and CM2.6, respectively. All authors discussed the results and commented on the manuscript.

## Additional information

**How to cite this article:** Biastoch, A. *et al*. Atlantic Multi-decadal Oscillation covaries with Agulhas leakage. *Nat. Commun.* 6:10082 doi: 10.1038/ncomms10082 (2015).

## Supplementary Material

Supplementary Information Supplementary Figures 1-6
