# Supplementary material for: Atlantic multi-decadal oscillation covaries with Agulhas leakage
Source: Nat Commun. 2015 Dec 10;6:10082. doi: 10.1038/ncomms10082 (PMC4682045; doi:10.1038/ncomms10082)
Supplement: Supplementary Information — Supplementary Figures 1-6 [file ncomms10082-s1.pdf]

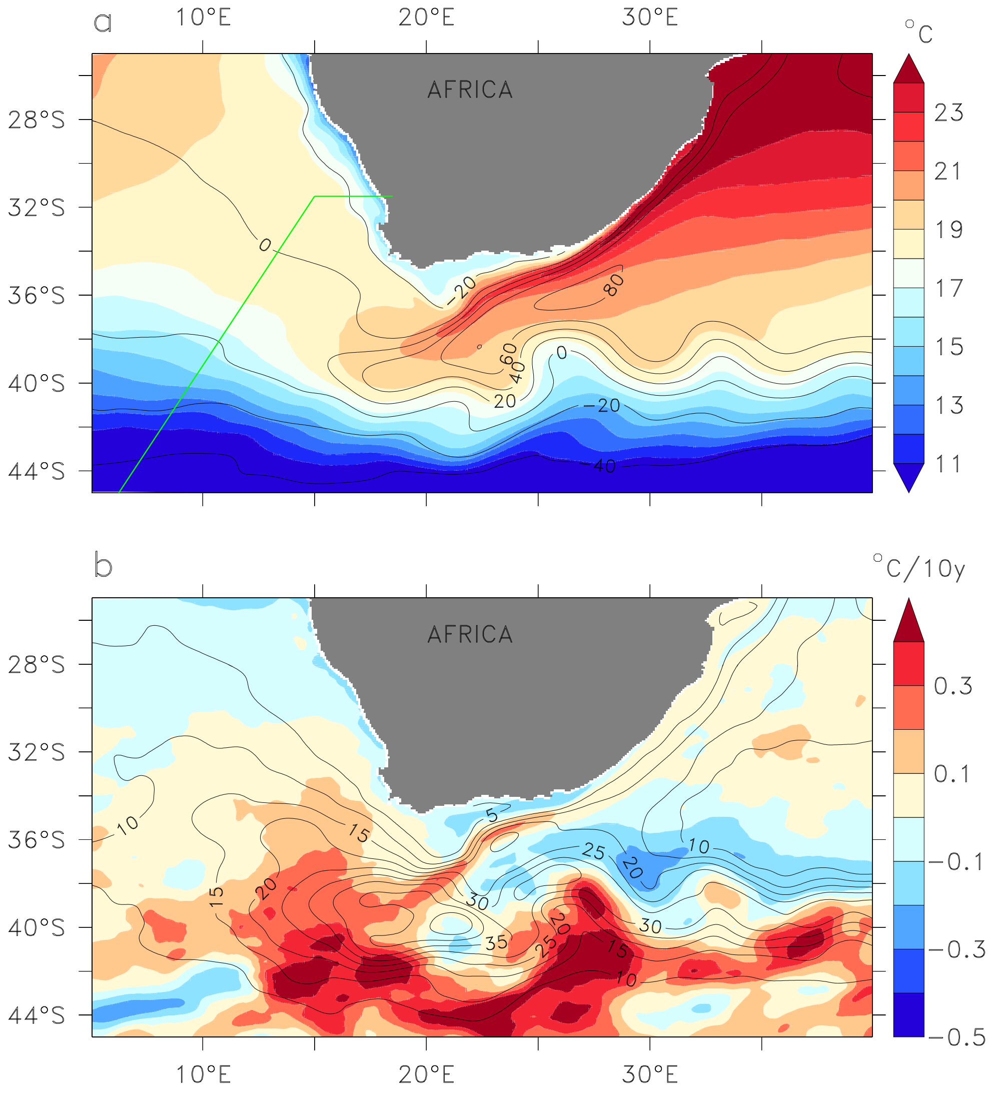

Supplementary Figure 1 | Similar to Fig. 1, but for the INALT01 model in the period 1965-2000. (**a**) Time-mean near-surface temperature (NST; colour) and SSH (contoured, in cm). (**b**) Linear NST trend (colour) and SSH standard deviation (contoured, in cm). The mean distributions of NST and SSH are well simulated in INALT01. Although the linear trend from the mid-1960s to the 2000s shows a finer detail compared to the observations (Fig. 1b), the general pattern is similar: a warming in the Agulhas Current (including its retroflection), the Cape Basin and the Agulhas ring corridor, and a cooling pattern in the southwest Indian Ocean and along the Agulhas Return Current.

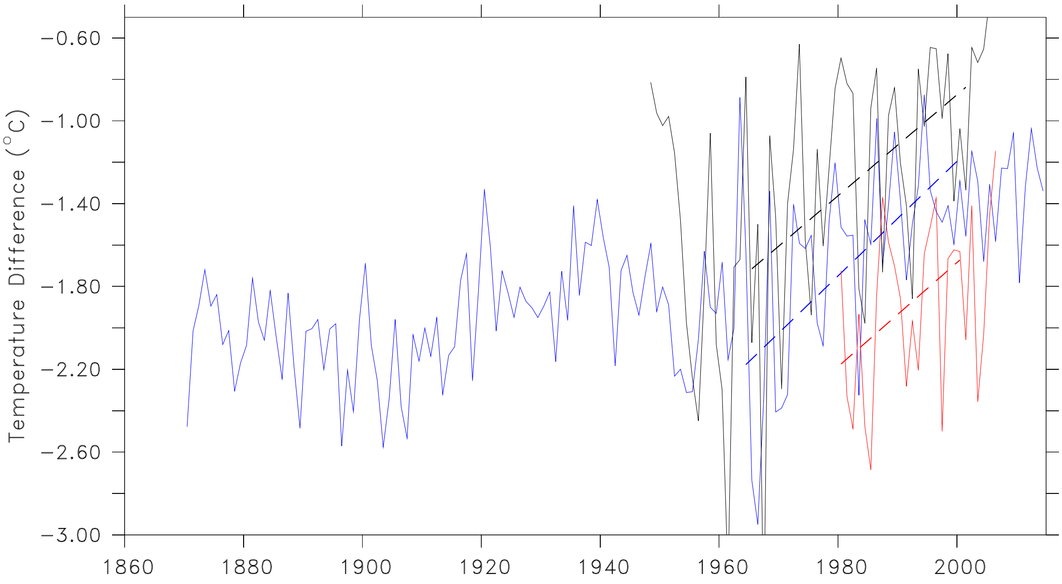

Supplementary Figure 2 | SST/NST difference between the Atlantic and Indian boxes (see Fig. 2) for HadISST (blue), INALT01 (black) and OFES (red). Trends (dashed) are significant at the 99% level for HadISST and INALT01 (shown for the period 1965-2000), and at the 95% level for OFES (shown for 1980-2000). Interannual std. dev. for the period 1965-2000 are 0.57 °C for INALT01, 0.39 °C for OFES (1980-2000) and 0.47 °C for HadISST (0.39 °C for the full 154 years). The temporal evolution of the temperature contrast between the Atlantic and Indian Oceans is masked by strong year-to-year

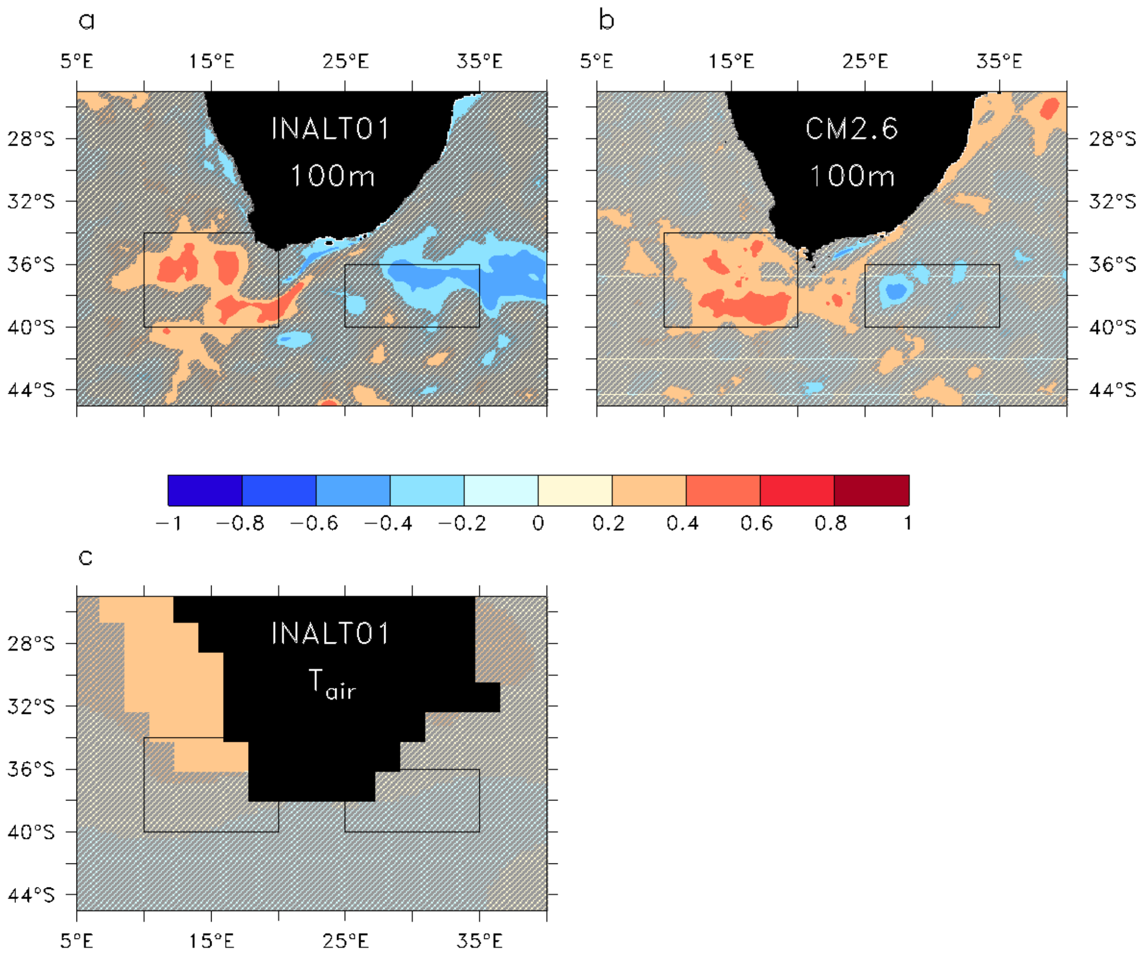

Supplementary Figure 3 | As Fig. 2, but correlation between annual Agulhas leakage and temperature at 100 m depth (**a**) for the forced-ocean model INALT01 and (**b**) for the coupled climate model CM2.6, as well as (**c**) with air temperature from reanalysis data[22] used as atmospheric forcing (and originally provided on a 2° grid) for INALT01 (regions hashed in grey denote significance below the 99% level). For the ocean models (INALT01, OFES), a near-surface temperature (NST) at ~15 m depth was chosen to prevent a direct imprint from the air temperature provided by the atmospheric boundary conditions through Bulk formulae. For the coupled climate model, sea surface temperature (SST) was used.

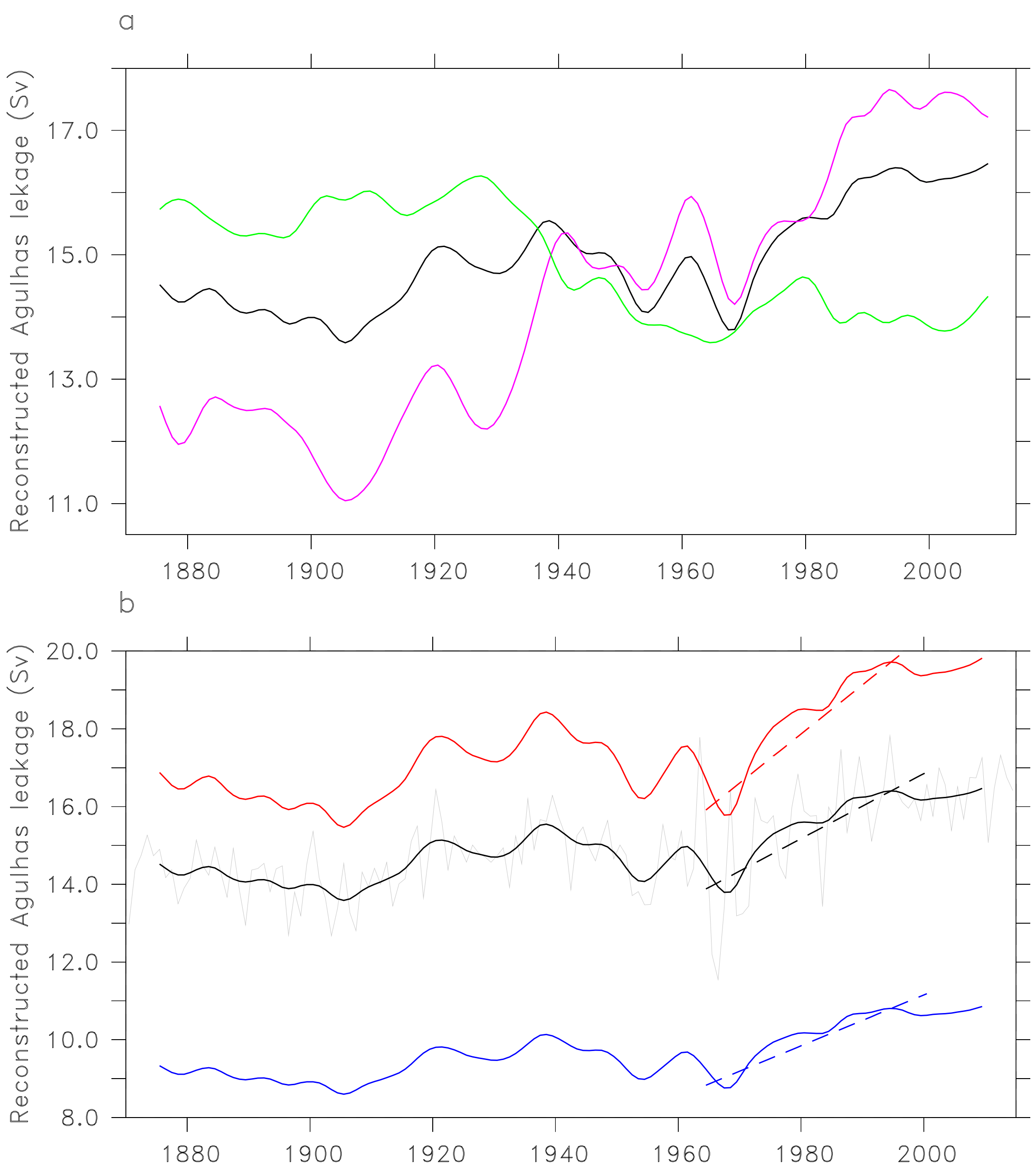

Supplementary Figure 4 | As Fig. 4, Agulhas leakage regressed from HadISST (**a**) using the INALT01 relation (black, decadally filtered) with fixed Indian Ocean NST (purple) and with fixed Atlantic Ocean NST (green), (**b**) using regressions based on INALT01 (black), OFES (red) and CM2.6 (blue). Slopes between 1965 and 2000 are 0.84 (INALT01), 1.26 (OFES) and 0.65 (CM2.6) Sv/decade (all significant at the 99% level).

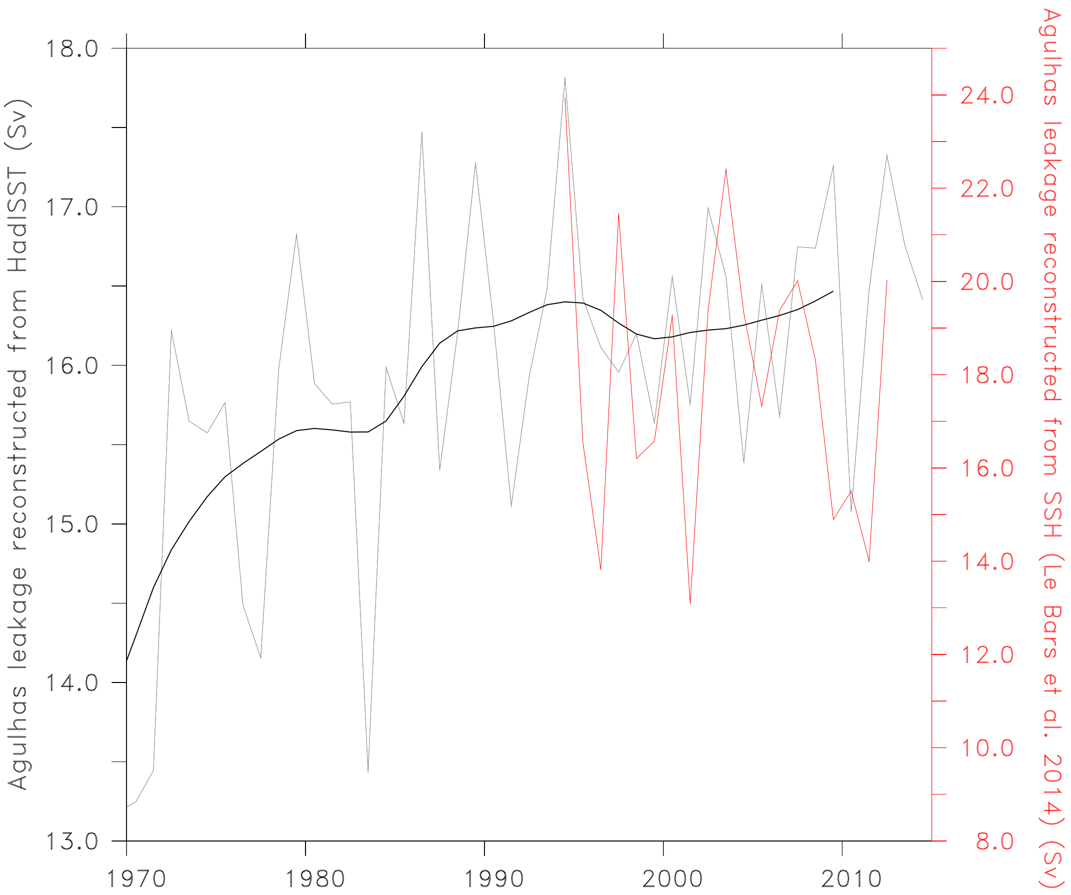

Supplementary Fig. 5 | As Fig. 4, annual (grey) and decadally filtered (black) Agulhas leakage regressed from HadISST. The red curve shows annual Agulhas leakage as derived through a dynamical criterion from SSH[11]. Both annual time series are weakly correlated (r = 0.4, significant at 90% confidence interval). Both methods have a different character, leading to a different focus in respect of timescales. Ref. 11 used a dynamical criterion for SSH, which compares transport estimates for the Agulhas Current and the Agulhas Return Current. The resulting differences is attributed to Agulhas leakage, a quantity that is (in particular because of the close distance of the measurements along the satellite track) valid at annual and higher temporal resolution; it is also difficult to calibrate in absolute transport numbers[11]. Our method involves the spreading of water masses, is verified also on annual timescales but

certainly much more targeted at interannual to decadal timescales (in particular because of the mixing processes taking place in the Cape Basin before reaching the Goodhope section). Nonetheless, both annual time series are weakly correlated for the overlapping period (r = 0.4, significant at 90% confidence interval).

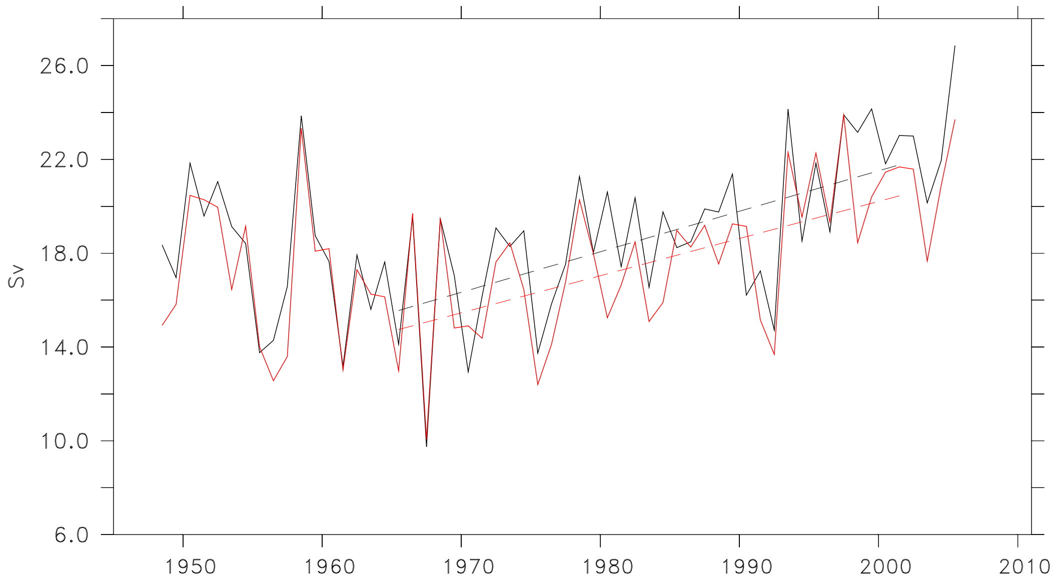

Supplementary Fig. 6 | Annual Agulhas leakage in INALT01, calculated from 5-daily mean (black) and from monthly mean (red) velocity averages. The linear trends over the period 1965-2000 (dashed lines) are comparable (1.7 Sv/decade for 5-daily, 1.6 Sv/decade for monthly data, all significant at the 99% level).
